# Protocol for a systematic review: Interventions addressing men, masculinities and gender equality in sexual and reproductive health: An evidence and gap map and systematic review of reviews

**DOI:** 10.1002/CL2.203

**Published:** 2018-08-31

**Authors:** Eimear Ruane‐McAteer, Jennifer Hanratty, Fiona Lynn, Esther Reid, Rajat Khosla, Avni Amin, Maria Lohan

## Background

It is globally recognised that protecting the health and rights of women and girls is central to development. There is also increasing recognition that men and boys can play a role as either supporting and championing or damaging and denying the health and rights of women and girls. Gender inequality including unequal gender norms related to masculinities and femininities is a key determinant of the health of men and women of all gender identities and sexualities, yet generally disproportionately disadvantages the opportunities and outcomes for women and girls, including in the particular field of sexual and reproductive health and rights (SRHR) (Gakidou et al., 2010; Kågesten et al., 2016).

Sexual and reproductive health and rights (SRHR) can be understood as the right for all, whether young or old, women, men or transgender, straight, gay, lesbian or bisexual, HIV positive or negative, to make choices regarding their own sexuality and reproduction, providing they respect the rights of others to bodily integrity. This definition also includes the right to access information and services needed to support these choices and optimize health (UNICEF, 2011; WHO, 2015).

The importance of addressing unequal gender norms, including harmful masculinities, and working with men and boys as well as with women and girls in relation to SRHR outcomes has gained traction in the international health and development policy and programme agenda. For example, a recent study funded by United States Agency for International Development analysed men's responses in Demographic and Health Surveys (DHS) in 33 low and middle income (LMIC) in order to examine whether men's involvement has an association with specific reproductive, maternal, and child health outcomes (Assaf and Davis, 2018). While the report concluded there were some positive associations, the study also highlighted the need for strengthened measures within DHS surveys to capture men's knowledge, attitudes, and behaviour related to reproductive, maternal, and child health. Overall, there are two primary drivers to this increased interest in researching the impact of the engagement of men and masculinities in SRHR outcomes. The first driver has been the shift in the global development paradigm from an overarching concern with population control in low‐resource countries to a human rights‐based approach aimed at empowering women to control their fertility and their access to safe childbearing. The step change which is notably attributed to the 1994 Cairo Conference on Population and Development and the report that followed (UNFPA, 1994) articulated a change in thinking which put gender equality at the heart of sexual and reproductive health. It also made explicit that the engagement of men in reproduction was heretofore largely ignored in the design of population and family planning policies and that this needed to change, especially to accelerate progress for women. For example, the United Nations Family Planning Report that followed spoke of the importance of educating men towards a ‘different interpretation of masculinity, replacing the one based on domination to one defined by shared responsibility’ (UNFPA, 1995, p. 16). This policy drive also came from over a decade of public attention to the HIV/AIDS pandemic in which men's sexual practices with men as with women came under greater critical enquiry (Sherr, 2010; Gutmann, 2011).

The second driver for an increased interest in masculinity and the engagement of men and boys has come from the work of feminists, including critical studies of men and masculinities, gender theorists and gender equality advocates working on health and development, and sexuality, reproduction and parenting. Their collective work has highlighted the importance of having efforts focused on empowerment of women and girls to be complemented with efforts to transform societal norms relating to gender (for example, Petchesky, 2003; Pullerwitz et al., 2010; Connell, 2012; Tallis, 2012; Agarawal, 2014; DFID PPA Learning Partnership Gender Group, 2015; Jewkes et al., 2015; Kabeer, 2015; Pearse and Connell, 2015). Their work explicitly acknowledges that transforming gender norms also requires working with men and boys to change their attitudes, behaviours and practices as well as changing patriarchal structures that perpetuate and uphold cultures of male privilege, power and entitlement (Lohan, 2007; WHO, 2007; Pullerwitz and Barker, 2008; Pullerwitz et al., 2010; Dworkin et al., 2013; Higgins et al., 2013; Agarawal, 2014; Kaufman et al., 2015). Included in the efforts of feminists to transform patriarchal norms and structures, are efforts to generate cultural shifts that promote norms about fatherhood that are premised on more active involvement of men in planning fatherhood and reproductive decision‐making, as well as childcare roles and responsibilities (Annandale and Clarke, 1996; Dudgeon and Inhorn, 2004; McAllister et al., 2012; Marsiglio et al., 2013; ILO, 2014; Lohan, 2015; Inhorn and Patrizio, 2015; Levtov et al, 2015; Promundo, 2016; Morrell et al., 2016).

Collectively, then, across international health and development policy and feminist scholarship more broadly, there is recognition of the need to have an approach which engages men alongside women in sexual and reproductive health and rights to achieve global health development goals for women and men, while not losing sight of addressing the structures of power and privilege that men hold as a group over women in society. However, the evidence on how best to engage men and address masculinities and what works and for what health outcomes, including those related to sexual and reproductive health, are variable (Dworkin et al.,2013).

One approach which is quickly gaining traction in international policy and practice (e.g. WHO, 2011; Greaves, 2014; DFID PPA, 2015); is a gender‐transformative inspired by Rao Gupta's speech at the 13th International AIDS conference in Durban in 2000 (Gupta, 2000). Gupta offered a continuum from least desirable to most desirable approaches to gender and development work: gender‐unequal (perpetuate gender inequalities), gender‐blind (ignore gender norms and conditions), gender‐sensitive (acknowledge but do not address gender inequalities), gender‐specific (acknowledge gender norms and consider women's and men's specific needs), gender‐transformative (create more gender‐equitable relationships), and gender‐empowering (empower women and men from the impact of destructive gender and sexual norms). Gupta's categories were adapted for example into a four‐category programming continuum: gender‐exploitative, gender‐neutral, gender‐sensitive, and gender‐transformative in a UNFPA & Promundo (2010) report with the latter gender transformative approach designed to encompass a gender‐empowering approach. The WHO also defines a gender‐transformative approach as one ‘that address the causes of gender‐based health inequities by including ways to transform harmful gender norms, roles and relations. The objective of such programmes is often to promote gender equality and foster progressive changes in power relationships between women and men’ (WHO, 2011: 78).

Hence, the term gender‐transformative programming or interventions has become increasingly germane as an approach that seeks to involve men and women of all different gender expressions and sexualities in efforts to improving gender equality and health, seeing both gender and health as inextricably linked. The approach explicitly goes beyond engaging and accommodating men in sexual and reproductive health to instead working with both men and women to foster critical examination of gender norms, to strengthen equitable gender norms and disrupt gender inequalities in order to improve SRHR outcomes for both men and women. A frequently cited example of a gender‐transformative intervention is Stepping Stones (Jewkes et al., 2010) and Stepping Stones and Creating Futures (Gibbs et al., 2017), both interventions designed to reduce intimate partner violence among men and women by empowering men and women to challenge forms of masculinities that support men's violence towards women.

While a gender transformative approach is quickly gaining traction in international policy and practice, the evidence for this approach and for which sexual and reproductive health needs to be assessed. We approach this analysis in a two‐stepped approach. First, the evidence and gap map will provide a pivotal overview of the entire body of systematic reviews on engaging men in SRHR outcomes, but specifically signalling out the quantity of available systematic reviews in the latter gender‐transformative category. This will be complemented by a deeper level systematic review of reviews in which we will evaluate the review evidence on a gender‐transformative approach to engaging with men in relation to SRHR outcomes (Hanratty et al., 2018). The choice of conducting a review of reviews was guided by the policy interest in deepening knowledge across the whole the range of WHO (2004) defined SRHR outcomes.

Our evidence and gap map is the first to explicitly map the entire range of systematic reviews of interventions engaging men in relation to SRHR outcomes. Our review of reviews will be the first to systematically synthesise the systematic review evidence on programmes that engage men and boys through a gender transformative approach to improve sexual and reproductive health and rights (SRHR) across all WHO (2004) defined outcomes.

## Policy relevance

This review will contribute to shaping a research agenda for addressing masculinities and challenging gender inequality in the context of sexual and reproductive health programmes. The SRHR outcomes examined in this review are drawn from the World Health Organization's Reproductive Health Strategy that was endorsed by Member States of the WHO in 2004 and will assist in driving this strategy forward. This strategy guides the work of the Department of Reproductive Health and Research including that of the Human Reproduction Programme (HRP) – a cosponsored programme within the UN system established to conduct research on sexual and reproductive health and rights.

The evidence and gap map generated from this review will provide researchers, programme planners and donors with a picture of all systematic reviews of evidence on engaging men and boys in SRHR interventions. The review of reviews will provide a more detailed analysis of the systematic review evidence on those interventions that specifically address masculinities from a gender transformative/gender equality perspective, the outcomes that are covered or not covered and the quality of this evidence. The review will help identify the gaps in research that need to be addressed and contribute to setting a research agenda for this area going forward.

## Objectives

To assess the state of the systematic review evidence on the effectiveness of interventions designed to engage men and boys in sexual and reproductive health and rights (SRHR) and to especially assess the systematic review evidence on gender‐transformative interventions.

The evidence review of reviews will focus on the following four questions:

**Review questions**

1. What is the state of the systematic review evidence on interventions designed to engage men and boys in sexual and reproductive health and rights?
2. What is the state of the systematic review evidence on interventions that are actively attempting to engage/ target men and boys *and* are explicitly designed to be gender‐transformative or promote gender‐equitable relationships to improve sexual and reproductive health and rights outcomes?
3. Which sexual and reproductive health outcomes are addressed in those reviews of evidence attempting to engage/ target men and boys and are explicitly designed to be gender‐transformative or address gender inequality to improve sexual and reproductive health outcomes?^1^
4. What is the methodological quality of the systematic reviews of evidence of interventions attempting to engage/ target men and boys and are explicitly designed to be gender‐transformative to improve sexual and reproductive health outcomes?

The analysis of findings will be addressed in two stages. In the first, we will produce a broad evidence and gap map summarising the existing systematic reviews of SRHR interventions involving men and boys including what types of approaches and what types of SRHR outcomes are covered. The second stage will be to produce a narrative synthesis of a subset of these systematic reviews of SRHR interventions attempting to engage men and boys that also seek to specifically gender‐transformative by addressing harmful masculinities or gender inequality. In the second stage, we will also present a summary of the quality appraisal of this evidence on gender‐transformative interventions that engage men and boys to improve SRHR outcomes.

## Existing reviews

There are several reviews that have looked at a gender‐transformative approach to the engagement of men and boys in relation to specific SRHR outcomes, such as HIV risk and violence (Dworkin et al., 2013) and maternal and child health (Kraft et al. 2014), as well as those addressing a gender‐transformative approach to the engagement of men and boys in relation to a broader set of SRHR outcomes (for example, WHO, 2007). In addition, the following evidence and gap maps exist pertaining to adolescent sexual health (3ie, 2015a), intimate partner violence (3ie, 2015b) and maternal and new‐born health (3ie, 2015c).

While there may be overlap between interventions included in these existing EGMs and reviews and our proposed work, based on our scoping searches, there are no other EGMs that have specifically looked at the engagement of men and boys in relation to SRHR outcomes and there are no other reviews of reviews that explicitly evaluate a gender‐transformative approach to the engagement of men and boys across all seven WHO sexual and reproductive health outcomes.

## Population

- Males aged 10‐60 years (inclusive)
- Men and adolescent boys of all sexual orientations and gender identities

Should a review also include studies with women only, data will only be extracted for those studies that also include men and boys.

## Intervention

Two categories of intervention will be eligible for this systematic review of reviews; the second category is a subset of the first.

1. Public health and educational interventions that are aimed at engaging men and boys in order to improve sexual and reproductive health.
2. Public health and educational interventions aimed at engaging men and boys and that explicitly address gender inequality to improve SRHR. This latter category is known as ‘gender‐transformative’ interventions (WHO, 2007).

### 1.1.1 Dimensions

Interventions in category 1 will be tabulated and summarised under broad characteristics of the interventions (e.g. geographic areas, populations included, intervention components and purported mechanisms of change) and outcomes targeted, categorised in line with the WHO Reproductive Health Strategy (see below). Interventions in category 2 (a subset of category 1) that explicitly seek to address gender inequality will then be narratively synthesised in detail (see outcome section below).

### 1.1.2 Intervention setting

Health and education services, including community, school and health facility settings

## Outcomes

### 1.1.3 Primary outcomes

We will include all systematic reviews of interventions that seek to engage men and boys with the aim of improving any of the seven sexual and reproductive health outcomes included in the WHO Reproductive Health Strategy (WHO 2004):

1. Helping people realize their desired family size (including: contraception and family planning; and the prevention and treatment of infertility)
2. Ensuring the health of pregnant women and girls and their new‐born infants (including: maternal and infant mortality; preventing complications in pregnancy, childbirth, and the postnatal period)
3. Preventing unsafe abortion
4. Promoting sexual health and well‐being (including: prevention of reproductive tract and sexually transmitted infections; HIV; and sexuality related human rights abuses e.g. sexual coercion (excluding conditions not acquired sexually e.g. testicular and prostate cancers, and more general men's health conditions)
5. Sexual and reproductive health in disease outbreaks (including: prevention of sexual transmission of Zika and Ebola viruses (evidence suggests virus can remain for many months in semen, amniotic fluid, and breastmilk))
6. Healthy adolescence for a healthy future (including improving sexual and reproductive health and education services; preventing unplanned pregnancy, unsafe sex (preventing STI/HIV), and unsafe abortion; harmful traditional practices e.g. female genital mutilation/cutting (FGM/C), child, early, and forced marriage; and sexual coercion and intimate partner violence (IPV))
7. Preventing and responding to violence against women and girls (including: intimate partner violence (IPV); sexual violence) and harmful practices (i.e. FGM; child, early, and forced marriage).

Specific outcomes will relate to improved gender equality, for example as measured through the GEM scale (Pullerwitz and Barker, 2008); as well as sub‐domains of the above outcome areas, for example, biological measures of improved SRHR such as reduction in STIs including HIV; early teenage pregnancies; early teenage births or infertility. Socio‐legal measures of reductions in child marriage or a reduction of rates of intimate partner violence are also relevant.

## Study designs

Systematic reviews synthesising findings from interventions of effect (RCT/quasi‐experimental) targeting sexual or reproductive health that aimed to engage men and/or boys can be included. The choice of conducting a review of reviews rather than primary intervention studies was guided by the necessity of including the broad scope of all seven WHO defined SRHR outcomes. We anticipate the number of reviews to run to several hundred reviews. We acknowledge that a limitation of a review of reviews is that the evidence is then limited to the interventions presented in reviews and will not cover more recent studies of interventions which have not yet been included in reviews up to 2018. The choice to include reviews of evidence of RCTs and quasi‐experimental studies only was informed by the need to evaluate high‐quality evidence on the effectiveness of gender‐transformative interventions.

Should a review include additional non‐experimental studies, data will only be extracted for experimental studies. Should the systematic review fail to present experimental and non‐experimental results separately, the review will not be included in the narrative synthesis but will be included in the evidence and gap map.

A review will be considered systematic when it contains a systematic search. A systematic search will be based on the reporting of a pre‐determined search strategy, specifying the location of the search, and stating the numbers and reasons for excluding papers from the final synthesis (e.g. PRISMA flow chart). Any disagreement on what constitutes a systematic review will be discussed by the author team until an agreement is reached.

## Stakeholder engagement

The EGM and review of reviews is informed by WHO's Special Programme of Research on Human Reproduction's (HRP) Human Reproduction – Gender and Rights Advisory Panel or the GAP. This advisory panel has been in existence for more than 20 years and is an external independent panel of experts on gender equality and human rights issues in relation to sexual and reproductive health. The GAP meets annually and reviews projects and provides critical guidance and feedback. In 2016 and 2017, the initial concept for the review of reviews was presented to the GAP who provided feedback on the framing and recommended a set of outputs that would be valuable to the advancing of the field of sexual and reproductive health in relation to engagement of men and boys. The GAP identified the need for synthesizing the evidence on masculinities and SRHR and assessing research gaps in order to inform a future agenda for research on this issue.

GAP is chaired by two leading experts – Dr Pascale Allotey (Director of the United Nations University International Institute for Global Health in Malaysia) and Dr Carmen Barosso, who also chairs the UN SG's independent accountability panel for the global strategy on women, children and adolescents' health. Other experts in the field of masculinity and gender equality who comprise the GAP include: Dr Gita Sen, founder of DAWN (Development Alternatives with Women for a New Era – a feminist network from the global south) and professor on gender and health equity at the Indian Institute of Management; Dr Emma Fulu, Director, Equality Institute, and lead author of numerous publications on men and violence against women prevention; and Dr Oswaldo Montoya, Secretariat of the MenEngage Alliance (a network of NGOs working on masculinities and health). The GAP's Secretariat is managed by Dr Avni Amin, staff member of WHO/HRP who is also a co‐author of this review.

## Searching for reviews

### 1.1.5 Search dates

We will search for reviews produced between: 2007‐2018. The starting point of the review marks twelve years since the United Nations International Conference on Population and Development in Cairo (1994). This was a watershed conference in shifting the broad approaches to improving sexual and reproductive rights globally and in making gender equality and the engagement of men intrinsic to the human rights approach. Our choice of start date is informed by the need to allow for gender‐transformative intervention studies and subsequent reviews of evidence of this approach to have materialised post this important conference. The review also begins at the end search date for a significant previous WHO review of the evidence (WHO 2007). This review will be included in our review of reviews. This review is internationally recognised by our stakeholders group to be the first review of a gender‐transformative approach to engaging men and boys in sexual and reproductive health.

### 1.1.6 Search terms

Search terms related to SRHR were adapted from Warren et al (2015) with the addition of “maternal mortality”,“forced sex” “sexual slavery”, “sexual exploitation” “coercive control”, “child prostitut*”, “child trafficking”, “trafficking of child*”, “female genital mutilation”, “FGM”, “female genital cutting”, “FGMC”, “female circumcis*”, “fertile*”, “infertil*”, (early and marriage), (child and marriage), (forced and marriage), (arranged and marriage), (abduction and marriage). Terms related to FGM and child marriage were adapted from Greene et al (2015) and Karumbi et al (2017). A number of more generic terms not specifically related to SRHR were removed from Warren et al (2015) string (e.g. “violence” “physical assault”).

Terms related to males and masculinities were developed and tested in a number of databases to ensure they captured all relevant papers. An edited Pearl Harvesting approach to searching databases for systematic reviews will be utilised to identify systematic review papers (Sandieson, 2006). Two terms were removed from Sandieson's original Systematic Review search string due to them producing a large number of irrelevant articles (“qualitative synthesis” and “realist synthesis”). While the Pearl Harvesting approach produced a large number of search results, after testing a number of more simplified searches for systematic reviews it was found that a number of potentially relevant articles would be missed without it. Search terms related to trials were adapted from Cochrane approved guidance (Eady, Wilczynski & Haynes, 2008; Watson & Richardson, 1999). SRHR, men and masculinities, systematic review, and trial search strings were combined and tested in three key databases (Medline, PsycINFO, Embase) before final agreement for terms were reached.

**Table:** 

**Search terms**			
1 AND 2 AND 3 AND 4 (limited to Human; 2007‐present)			
**#1 SRHR**	**#2 Males/Masculinities**	**#3 Systematic Review**	**#4 Trials**
sexual health or reproductive health or maternal health or maternal welfare or maternal mortality or neonatal health or perinatal care or perinatal health or prenatal care or prenatal health or antenatal health or ante‐natal health or postnatal health or post‐natal health or post‐part* or post part* or newborn health or family planning or contracepti* or condoms or condom or pregnan* or abortion or induced abortion or abort* or birth or miscarriage or spontaneous abortion or stillb* or Minimum Initial Service Package or obstetric* or gynecology or gynaecology or safe motherhood or safe delivery or skilled birth attend* or sexually transmitted infection* or sexually transmitted disease* or HIV or Human immunodeficiency virus or AIDS or acquired immune deficiency syndrome or PMTCT or rectovaginal fistula or urethra fistula or urinary tract fistula or genital trauma or genital injury or vaginal trauma or vaginal injury or gender‐based violence or gender based violence or partner violence or family violence or violence against women or domestic violence or sexual abuse or sex crime or sexual crime or domestic violence or sexual violence or rape or intimate partner violence or partner violence or partner abuse or sexual assault or sexual harassment or sexual coercion or forced sex or sexual slavery or sexual exploitation or coercive control or child prostitut* or child trafficking or trafficking of child* or female genital mutilation or FGM or female genital cutting or FGMC or female circumcis* or fertile* or infertil* or (early and marriage) or (child and marriage) or (forced and marriage) or (arranged and marriage) or (abduction and marriage)	men or man or male or males or boy or boys or masculin* or father* or gender or equality	“data synthesis” or “evidence synthesis” or metasynthesis or meta‐synthesis or “narrative synthesis” or “quantitative synthesis” or “research synthesis” or “synthesis of evidence” or “thematic synthesis” or metaanaly* or meta‐analy* or metaanalysis or meta‐analysis or systematic or “systematic map*” or “systematic overview*” or “systematic review*” or “systematically review*” or “bibliographic search” or “database search” or “electronic search” or handsearch* or “hand search*” or “keyword search” or “literature search” or “search term*” or “article reviews” or “literature review” or “overview of reviews” or “review literature” or “reviewed the literature” or “reviews studies” or “this review” or “scoping stud*” or “overview study” or “overview of the literature” or meta‐ethnograph* or meta‐epidemiological or “data extraction” or “meta‐regression”	random* or trial or placebo or group or groups or intervention or interventions

### 1.1.7 Information sources

Peer‐reviewed literature: CINAHL; Medline; PsycINFO; Social Science Citation Index–expanded; Cochrane Library; Campbell Collaboration; Embase; Global health Library and Scopus.

Grey literature: A google search of the gray literature will be conducted using a condensed list of provided search terms.

The bibliographies of all identified gender transformative reviews will also subsequently searched for any outstanding reviews.

Report Characteristics: Reports will not be limited to English language.

### 1.1.8 Data screening

Records will be collated, and duplicates removed, using Endnote software. One author will then remove obviously irrelevant records. Two independent reviewers will apply the inclusion and exclusion criteria when screening titles, abstracts, and full‐text results for eligibility using Distiller Systematic Review Software. Disagreement or uncertainty surrounding inclusion of an article will be brought to the next screening stage and discussed and, where necessary, deferred to a third reviewer for a decision.

### 1.1.9 Data extraction

A pilot data extraction form will be agreed in advance and piloted on 10 cases and reviewed prior to being applied to the remainder of the included articles. Double‐blind data extraction will be conducted by two authors. At a minimum, we will extract the following details from the included reviews; population, intervention/topic, comparison, outcomes, geographic locations. For gender‐transformative reviews we will also extract more detailed outcomes, key components and theoretical rationale of interventions included.

### 1.1.10 Data quality assessment

We will formally assess the quality of only the systematic reviews of gender‐transformative interventions only. The AMSTAR tool will be used to assess the methodological quality of these systematic reviews. Double‐blind data extraction will be conducted by two authors and any discrepancies resolved through discussion with a third reviewer.

## Evidence synthesis

Review question one will be addressed through the creation of a broad evidence and gap map. This broad evidence and gap map will provide a visual, interactive summary of the existing systematic reviews of impact evaluations of interventions involving men to improve SRHR. We will also summarise the characteristics of the evidence base by geographic location, types of outcomes reviewed, intervention focus/ topic.

Review questions two, three and four will be addressed with a more detailed narrative synthesis of those reviews on gender‐transformative studies that sought to engage men and/or boys and address gender inequality in the seven sexual and reproductive health domains outlined above and derived from the WHO Reproductive Health Strategy (WHO 2004).

In relation to question three, we will address overlap of gender‐transformative studies, which is where certain primary studies may appear more than once across reviews as suggested by Polanin et al. (2017). We will do so by examining the number of individual studies of an intervention, e.g. Stepping Stones (Jewkes et al., 2010), as well as the number of times each study may appear across reviews. We will also define the characteristics of the interventions that reviews note have been especially effective, in what context and why, along with those which were especially ineffective or harmful, for example, by leading to an increase in early child marriage or an increase in inequality between men and women.

## Review authors

**Lead review author:** The lead author is the person who develops and co‐ordinates the review team, discusses and assigns roles for individual members of the review team, liaises with the editorial base and takes responsibility for the on‐going updates of the review.

Maria Lohan is lead and corresponding author.

**Table:** 

**Name:**	**Professor Maria Lohan**
Title:	Chair in Social Science & Health & Director of Research
Affiliation:	School of Nursing and Midwifery, Queen's University Belfast
Address:	97 Lisburn Road Medical Biology Centre
City, State, Province or County:	Belfast
Post code:	BT9 7BL
Country:	United Kingdom
Phone:	+44 28 9097 2233
Email:	m.lohan@qub.ac.uk
**Co‐author(s):**	
**Name:**	**Dr Eimear Ruane McAteer**
Title:	Research Fellow
Affiliation:	School of Nursing and Midwifery, Queen's University Belfast
Address:	97 Lisburn Road Medical Biology Centre
City, State, Province or County:	Belfast
Post code:	BT9 7BL
Country:	United Kingdom
Phone:	+44 28 9097 2233
Email:	E.Ruane-McAteer@qub.ac.uk
**Name:**	**Dr Jennifer Hanratty**
Title:	Research Fellow
Affiliation:	Campbell UK and Ireland, Queen's University Belfast
Address:	6 College Green
City, State, Province or County:	Belfast
Post code:	BT7 1LN
Country:	United Kingdom
Phone:	+44 28 9097 2593
Email:	j.hanratty@qub.ac.uk
**Name:**	**Dr Fiona Lynn**
Title:	Lecturer
Affiliation:	School of Nursing and Midwifery, Queen's University Belfast
Address:	97 Lisburn Road Medical Biology Centre
City, State, Province or County:	Belfast
Post code:	BT9 7BL
Country:	United Kingdom
Phone:	+44 28 9097 5784
Email:	f.lynn@qub.ac.uk
	** **
**Name:**	**Dr Esther Reid**
Title:	Lecturer (Education)
Affiliation:	School of Nursing and Midwifery, Queen's University Belfast
Address:	97 Lisburn Road Medical Biology Centre
City, State, Province or County:	Belfast
Post code:	BT9 7BL
Country:	United Kingdom
Phone:	+44 28 9097 2259
Email:	Esther.Reid@qub.ac.uk
**Name:**	**Dr Avni Amin**
Title:	Technical Officer
Affiliation:	World Health Organization
Address:	Avenue Appia 20
City, State, Province or County:	Geneva
Post code:	1202
Country:	Switzerland
Phone:	+41‐22‐7912111
Email:	amina@who.int
**Name:**	**Rajat Khosla**
Title:	Human Rights Adviser
Affiliation:	World Health Organization
Address:	Avenue Appia 20
City, State, Province or County:	Geneva
Post code:	1202
Country:	Switzerland
Phone:	+41‐22‐7912111
Email:	amina@who.int

## Roles and responsibilities

- Content: Professor Maria Lohan, Dr Eimear Ruane‐McAteer, Dr Fiona Lynn, Dr Esther Reid
- Systematic review methods: Dr Jennifer Hanratty, Dr Fiona Lynn, Dr Eimear Ruane‐McAteer
- Statistical analysis: We do not intend to conduct meta‐analysis. Dr Hanratty and Dr Lynn will provide expertise on interpretation of the findings of the included systematic reviews where necessary.
- Information retrieval: Dr Jennifer Hanratty, Dr Fiona Lynn

### 1.1.11 Roles

1. Professor Maria Lohan, Chair in Social Science and Health and expert in engaging men and boys in sexual and reproductive health and systematic reviews.
2. Dr Eimear Ruane‐McAteer, Research Fellow and expert in men's health with previous experience of conducting systematic reviews
3. Dr Fiona Lynn, Health Economist and expert in maternal and child health and systematic reviews, including meta‐ analysis
4. Dr Esther Reid, Lecturer in Midwifery and expert in maternal and child health in low‐resource settings as well as systematic reviews
5. Dr Jennifer Hanratty, Research Fellow, and expert in Systematic Reviews, Campbell Collaboration UK & Ireland Centre for Evidence and Social Innovation
6. Dr Avni Amin, Technical Officer, Department of Reproductive Health and Research, World Health Organization, focal point for gender equality, masculinities and expert in violence against women and girls
7. Mr Rajat Khosla, Human Rights Advisor, Department of Reproductive Health and Research, World Health Organization, focal point for human rights.

### 1.1.12 Responsibilities

Professor Lohan will manage all aspects of this study design and study processes. Dr Ruane‐McAteer will be employed full‐time on the study to undertake searches, help refine protocol, and undertake data extraction, data appraisal and data synthesis. Professor Lohan and Dr Reid will share the double‐blind processes of data extraction, data appraisal and data analysis with Dr Ruane‐McAteer. Dr Lynn will contribute to study design, review quality assessment, risk of bias assessments and narrative synthesis. Dr Hanratty will offer additional methodological advice. Dr Ruane‐McAteer and Professor Lohan will draft review synthesis and all other team members will comment on and edit manuscript. The team will meet weekly and tasks will be assigned and monitored on a weekly basis. WHO staff – Dr Avni Amin and Rajat Khosla will provide overall direction on the review including research questions, protocol, findings of the review and drafts of the papers coming out of the review. They will also identify external experts through a technical advisory group to provide peer review feedback to this review. Involvement by WHO and the external advisory group will occur through scheduled teleconferences and by reviewing drafts of all written materials.

## Funding

This review is funded by the Human Reproduction Programme (UNDP/ UNFPA / UNICEF/ WHO/ World Bank Special programme of research, development and research training in human reproduction – HRP) at the World Health Organization (WHO).

## Potential conflicts of interest

The authors have no conflict of interest to report. However, one of the authors, ML was involved in developing a relevant intervention, which could be included in a systematic review.

## Preliminary timeframe

The time frame for the study will be February 1^st^ 2018 to February 2019 (12 months).

**Table:** 

**Date**	**Task**
February	Scope review for protocol development
March	Present and agree protocol with WHO March15^th^ and submit TRF to Campbell Collaboration by March 30^th^
April‐ September	Submit protocol to Campbell 30^th^ April 2018. Conduct searches, article screening, data extraction and quality assessment
September to December	Develop data synthesis
December	Write up of report for WHO
February 2019	Submit review for publication

- Date you plan to submit a draft protocol: 30 April 2018
- Date you plan to submit a draft review: February, 2019.
